# Cloning and Molecular Characterization of the *phl*D Gene Involved in the Biosynthesis of “Phloroglucinol”, a Compound with Antibiotic Properties from Plant Growth Promoting Bacteria *Pseudomonas* spp.

**DOI:** 10.3390/antibiotics12020260

**Published:** 2023-01-28

**Authors:** Payal Gupta, Prasanta K. Dash, Tenkabailu Dharmanna Sanjay, Sharat Kumar Pradhan, Rohini Sreevathsa, Rhitu Rai

**Affiliations:** 1 ICAR-National Institute for Plant Biotechnology, Pusa Campus, New Delhi 110012, India; 2 ICAR-National Rice Research Institute, Cuttack 753006, India; 3 Indian Council of Agriculture Research, Krishi Bhawan, New Delhi 110001, India

**Keywords:** phloroglucinol, polyketide, DAPG, *phl*D, Ramachandran plot, PMDB

## Abstract

*phl*D is a novel kind of polyketide synthase involved in the biosynthesis of non-volatile metabolite phloroglucinol by iteratively condensing and cyclizing three molecules of malonyl-CoA as substrate. Phloroglucinol or 2,4-diacetylphloroglucinol (DAPG) is an ecologically important rhizospheric antibiotic produced by pseudomonads; it exhibits broad spectrum anti-bacterial and anti-fungal properties, leading to disease suppression in the rhizosphere. Additionally, DAPG triggers systemic resistance in plants, stimulates root exudation, as well as induces phyto-enhancing activities in other rhizobacteria. Here, we report the cloning and analysis of the *phl*D gene from soil-borne gram-negative bacteria—*Pseudomonas*. The full-length *phl*D gene (from 1078 nucleotides) was successfully cloned and the structural details of the PHLD protein were analyzed in-depth via a three-dimensional topology and a refined three-dimensional model for the PHLD protein was predicted. Additionally, the stereochemical properties of the PHLD protein were analyzed by the Ramachandran plot, based on which, 94.3% of residues fell in the favored region and 5.7% in the allowed region. The generated model was validated by secondary structure prediction using PDBsum. The present study aimed to clone and characterize the DAPG-producing *phl*D gene to be deployed in the development of broad-spectrum biopesticides for the biocontrol of rhizospheric pathogens.

## 1. Introduction

Plant growth-promoting rhizobacteria (PGPR) are bacteria that colonize some or all parts of the rhizosphere environment and have the capability to promote plant growth [1,2,3] either directly by antibiosis or indirectly by quorum sensing. PGPR produce non-volatile metabolites that can directly stimulate plant growth, inhibit plant pathogens, and/or induce host–defense mechanisms against pathogens [4,5]. *Pseudomonas fluorescens* is an important group of PGPR that suppress root and seedling diseases by producing non-volatile secondary metabolite phloroglucinol. Genetic methods [6,7,8] and direct isolation from the soils of diseased plants [9,10,11] have shown the importance of DAPG and its derivatives as biocontrol activity agents. These compounds act as antibiotics, antimicrobials or antifungals, signaling molecules, and pathogenicity factors. Several antibacterial and antifungal compounds from plants have been characterized [12] and their mechanisms of action have been delineated [13]; biosynthesis and genetic regulation of DAPG in the *Pseudomonas* spp. have been the focus of active research.

**Figure 1:**
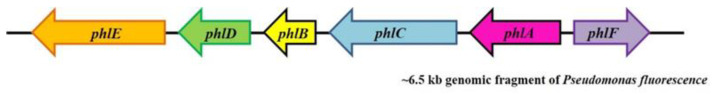
**Schematic representation of a** ~6.5 kb genomic fragment of *Pseudomonas* harboring the genes responsible for the biosynthesis of 2,4-DAPG by *phl* operon. *phl* operon comprises four genes *phl*A, *phl*B, *phl*C, and *phl*D. The operon is flanked on either side by *phl*E and *phl*F genes that are separately transcribed and coded for the putative efflux and regulatory (repressor) proteins, respectively. They are not required for phloroglucinol production.

Most of the genes required for the biosynthesis of DAPG have been cloned and characterized from different strains of *Pseudomonas*. These genes are recognized as conserved in 2,4-DAPG, producing pseudomonads that have been isolated from various soil samples collected from around the world [14,15,16]. All of the genes are arranged as an operon on the *phl* locus, spanning a genomic fragment of ~6.5 kb, comprising six genes, viz. *phl*A, *phl*B, *phl*C, *phl*D, *phl*E, and *phl*F. While *phl*A, *phl*B, *phl*C, and *phl*D are transcribed as an operon; they are flanked on either side by *phl*E and *phl*F. *phl*E codes for an efflux protein and *phl*F encodes a repressor protein (Figure 1).

The DAPG gene cluster is self-sufficient for the biosynthesis and regulation of 2,4-diacetylphloroglucinol. Amongst all genes, *phl*D is the key gene responsible for the production of (MAPG), while, *phl*A, *phl*B, and *phl*C are necessary to convert MAPG to 2,4-DAPG. Products of these genes resemble neither type I nor type II PKS enzyme systems. Rather, *Phl*D shows similarity to plant chalcone synthases, indicating that phloroglucinol synthesis is mediated by a novel kind of PKS [17,18,19]. 

Apart from changes in gene expression, the production of 2,4-DAPG in many strains of fluorescent *Pseudomonas* spp. is stimulated by physical factors, such as a concentration of glucose [20] or concentrations of sucrose/ethanol [19,21]. Moreover, zinc sulfate and ammonium molybdate have been reported to favor 2,4-DAPG production in some strains, whereas inorganic phosphate in general has an inhibitory effect [20]. 

*phl*D gene, responsible for the bio-synthesis of MAPG, shows similarity to novel type III polyketide synthase (PKS) [22]. *phl*D iteratively condenses three molecules of malonyl-CoA that subsequently cyclize [23] to form mono-acetyl phloroglucinol (MAPG). However, it is delineated that *Phl*D catalyzes the condensation of three molecules of malonyl-CoA into 3,5-diketoheptanedioate [24] and this polyketide intermediate through decarboxylation/cyclization, forms phloroglucinol [25].

Our laboratory has isolated and cloned and characterized *phl*A, *phl*B, and *phl*C; the downstream genes [26,27,28] of *phl* operon, and the current study focuses on the cloning and characterization of the *phl*D gene, an upstream/first committed step of the *phl* operon of *Pseudomonas* and its in-depth characterization to obtain deep insight into the PHLD function. This will help in fine-tuning (upregulating/downregulating) the bio-synthesis of 2,4-DAPG in response to potent fungal and bacterial pathogens for improving the biocontrols of plant pathogens.

## 2. Materials and Methods

### 2.1. Genomic DNA Isolation and PCR Amplification of the phlD Gene

Genomic DNA of the *Pseudomonas* spp. strain RS9 (KP057506) [29] was isolated as described earlier [26]. The *phl*D gene from *Pseudomonas* spp. was amplified by the polymerase chain reaction-based strategy. The forward and reverse primers were designed using the PRIMER 3 tool, viz, *phl*D (FP): CCGACTAGTAGGACTTGTCATGTCTACTCTTTG and *phl*D (RP): GGAAAGCTTCGTGCAATGTGTTGGTCTGTCA were designed using the nucleotide sequence of *Pseudomonas fluorescens* (U41818) available in the EMBL database. Restriction sites for the enzymes *Spe*I and *Hind*III were incorporated at the 5′ ends of forward and reverse primers (underlined sequences), respectively. The PCR reaction mixture consisted of 10 pmol of each primer, 50 ng of template DNA, 50 mM KCl, 10 mM Tris-HCl (pH 9.0), 0.1% Triton, 2.5 mM MgCl_2_, 0.2 mM of each dNTP, and a 1.25 unit of Phusion *Taq* DNA polymerase in 100 μL of volume. The thermal cycling was performed with an initial denaturation cycle of 3 min at 98 °C, followed by 30 cycles of (i) denaturation at 98 °C for 20 s; (ii) annealing for 30 s at 55 °C; (iii) extension for 30 s at 72 °C, as well as one cycle of the final extension for 7 min at 72 °C. 

### 2.2. Cloning of the phlD Gene in pBluescript (SK+) Vector

For cloning of the PCR amplified *phl*D gene into the pBluescript (SK+) vector, the PCR product (insert) and pBluescript (SK+) vector DNA were double digested with *Spe*I and *Hind*III. The reaction mixture was incubated at 37 °C for 3 hours. Restricted DNA was gel-purified using a Zymo clean gel DNA recovery kit. Purified 50 ng of linearized pBluescript vector and 100 ng of a double-digested PCR product were ligated using T_4_ DNA ligase. The ligated mixture was incubated at 4 °C overnight for ligation and used for transformation into *E. coli.* The transformed colonies (white in color) obtained after overnight incubation at 37 °C were picked and streaked onto fresh LA-carbenicillin (100 µg/mL) plates. 

### 2.3. Confirmation of Cloning and Sequencing

Recombinant colonies were confirmed by restriction digestion with *Spe*I+*Hind*III enzymes. The restricted DNA samples were analyzed on 1.2% agarose gel. The complete nucleotide sequence was determined by the Sanger di-deoxy sequencing. M13F and M13R primers were used for sequencing. *phl*D gene-specific primers were also used for confirming the sequence. The final sequence was determined from both strands and a comparison of *phl*D nucleic acid and amino acid sequences with already existing sequences was performed. The deduced amino acid sequence of PHLD from *Pseudomonas* RS-9 was compared with type III PKS from gram-positive bacteria and other plants by a multiple sequence alignment using MAFFT version 7.271 program [30] with the L-INS-I strategy and output in Phylip format. A similarity score for each nucleotide of the aligned sequences was calculated by ESPRIPT 3.0 [31] (https://espript.ibcp.fr/ESPript/ESPript/, accessed on 20 February 2022) with default parameters. Conserved domain annotation analysis was performed using InterProScan [32].

### 2.4. Phylogenetic Analysis

For estimation of the phylogenetic relationship between PHLD from various Pseudomonas strains and type III PKS from gram-positive bacteria and plants, the amino acid sequences were retrieved from the NCBI database. A multiple sequence alignment for the respective amino acid sequences was performed by Clustal Omega [33] and an un-rooted tree was constructed in MEGA10 [34] using the maximum likelihood (ML) method. Tree topology was searched using the nearest neighbor interchanges (NNIs) algorithm [35]. The LG+G+I substitution model was employed. The gamma shape parameter was estimated directly from the data and the analysis was performed using 1000 bootstrap replicates. The proportion of invariable sites was fixed. The tree was obtained in the Newick format.

### 2.5. Structure Prediction

The model of the PHLD protein was predicted using the I-TASSER server (http://zhanglab.ccmb.med.umich.edu/I-TASSER/, accessed on 25 February 2022) [36]. I-TASSER (Iterative Threading ASSEmbly Refinement) is a hierarchical approach to protein structures and function prediction. Structural templates were first identified from PDB by the multiple threading approach, LOMETS; full-length atomic models were then constructed by iterative template fragment assembly simulations. The generated model was refined using ModRefiner (http://zhanglab.ccmb.med.umich.edu/ModRefiner/, accessed on 3 March 2022). ModRefiner is an algorithm for high-resolution protein structure refinement. Both side-chain and backbone atoms were completely flexible during structure refinement simulations. ModRefiner allowed the assignment of a second structure that was used as a reference to which the refinement simulations were driven. The ModRefiner was used to draw the initial starting model of PHLD closer to its native state.

### 2.6. Ramachandran Plot Analysis

The stereochemical properties of the PHLD protein were assessed by the Ramachandran plot analysis using RAMPAGE [37]. This allowed visualization of energetically allowed regions for backbone dihedral angles ψ against φ of amino acid residues in the PHLD protein structure. The residues in the disallowed region were further refined by using Modloop (https://modbase.compbio.ucsf.edu/modloop/, accessed on 5 March 2022). Modloop relies on MODELLER, which predicts the loop conformations of PHLD by the satisfaction of spatial restraints, without relying on a database of known protein structures [38]. 

### 2.7. Validation and Visualization of Modeled Structure

The validation of the modeled structure was performed using PDBsum [39] and PROCHECK [40]. Structure visualization was performed using PyMOL. The predicted model of the protein was submitted to the Protein Model Database [41] (http://srv00.recas.ba.infn.it/PMDB/main.php, accessed on 10 March 2022).

## 3. Results

**Figure 2:**
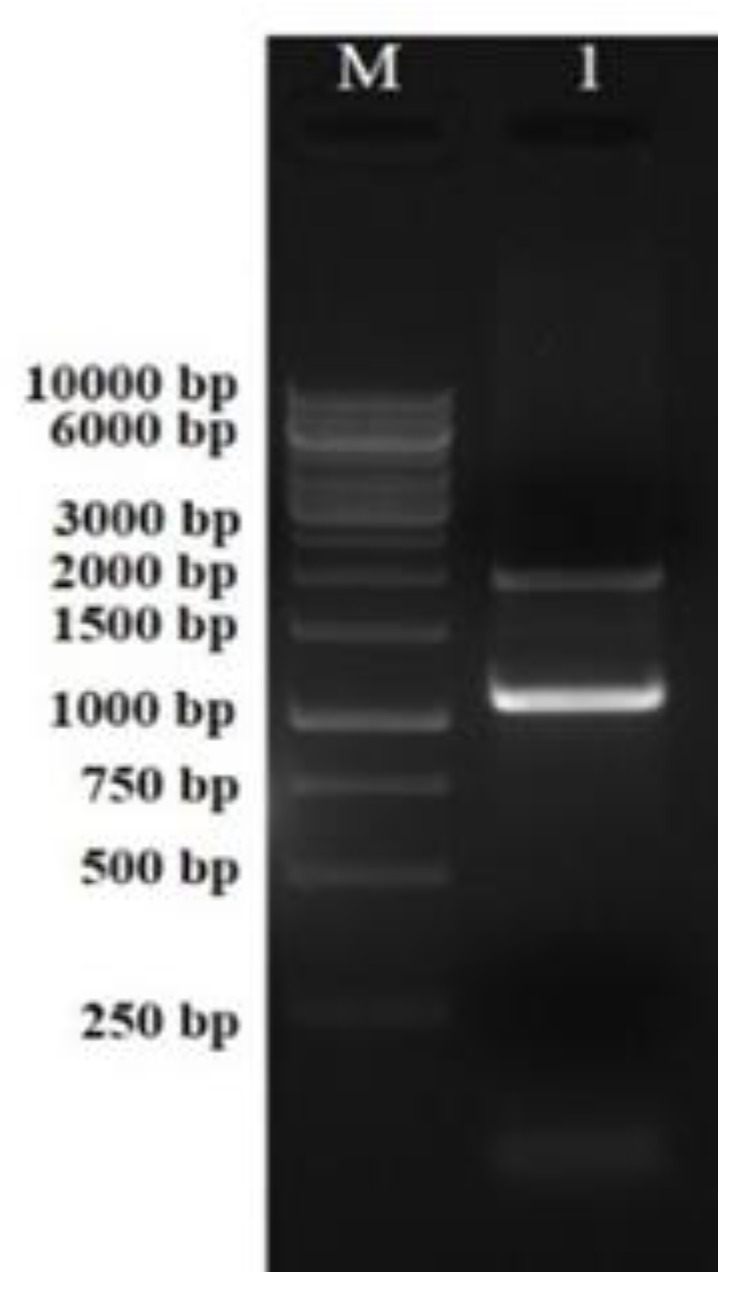
PCR amplification of the *phl*D gene from genomic DNA of *Pseudomonas fluorescens.* Lane M: 1 kb DNA ladder; Lane 1: ~1078 kb amplicon of the *phl*D gene.

**Figure 3:**
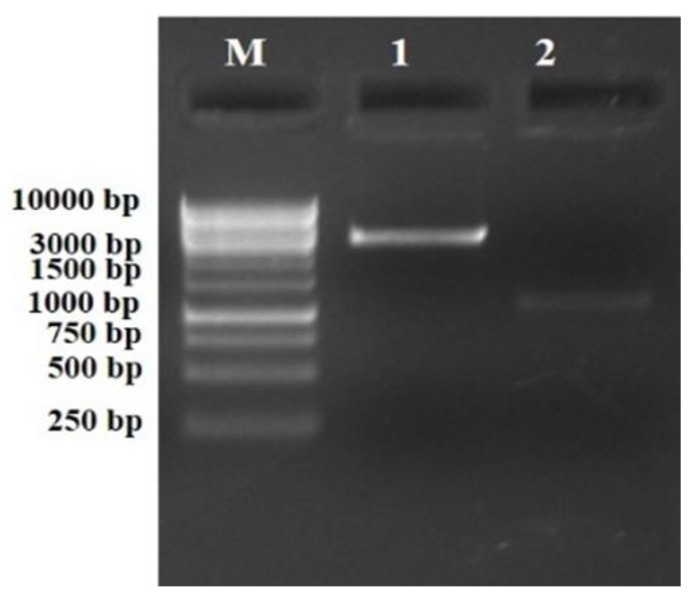
Restriction profile of the double-digested PCR product and control pBluescript vector. Lane M: 1 kb DNA ladder. Lane 1: ~3 kb fragment of pBS digested with *Spe*I+*Hind*III. Lane 2: ~1.078 kb fragment of purified PCR product. The PCR product was digested with *Spe*I+*Hind*III.

PCR amplification of the *phl*D gene from the genomic DNA of the *Pseudomonas* spp. strain RS9 (KP057506) [29] resulted in a fragment of 1 kb (Figure 2). The amplified PCR product and pBluescript control vector were then restricted with *Spe*I and *Hind*III restriction enzymes. This resulted in a 1 kb fragment PCR product with sticky ends (insert) and 3 kb of linearized control vector pBluescript (SK+) with sticky ends for *Spe*I and *Hind*III (Figure 3). The purified double-digested PCR product (insert) was ligated into the linearized pBluescript vector.

**Figure 4:**
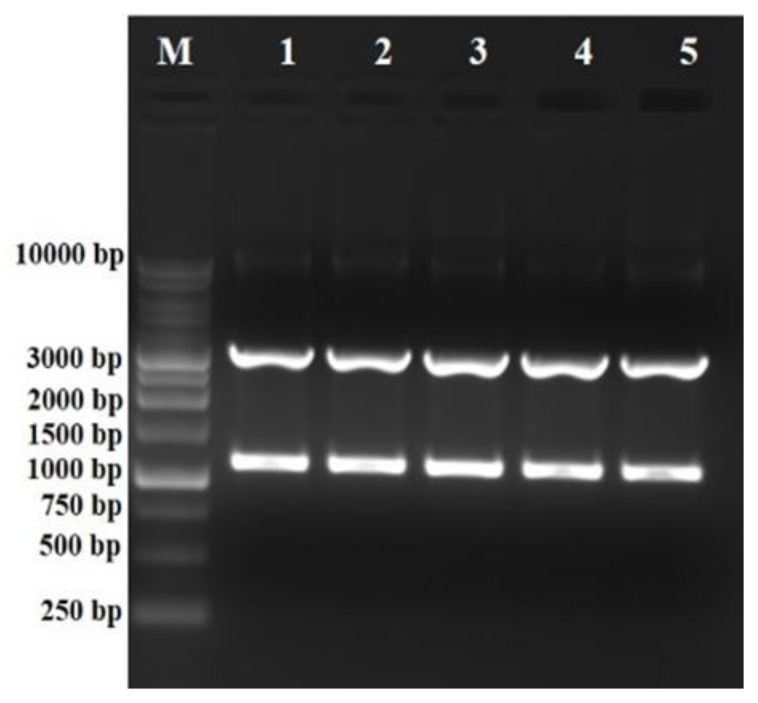
Screening and confirmation of positive putative clones by restriction digestion, respectively. Lanes 1–5: restriction digestion with *Spe*I+*Hind*III released a fragment of ~1.078 kb of the *phlD* gene and ~3 kb fragment of pBluescript (SK+) vector backbone.

The ligated mixture was transformed into *E. coli* DH-5α competent cells and five randomly picked white colonies were used for the plasmid isolation. The presence of the *phl*D gene was confirmed by restriction digestion with *Spe*I+*Hind*III enzymes that released the expected fragment of ~1 kb (Figure 4).

**Figure 5:**
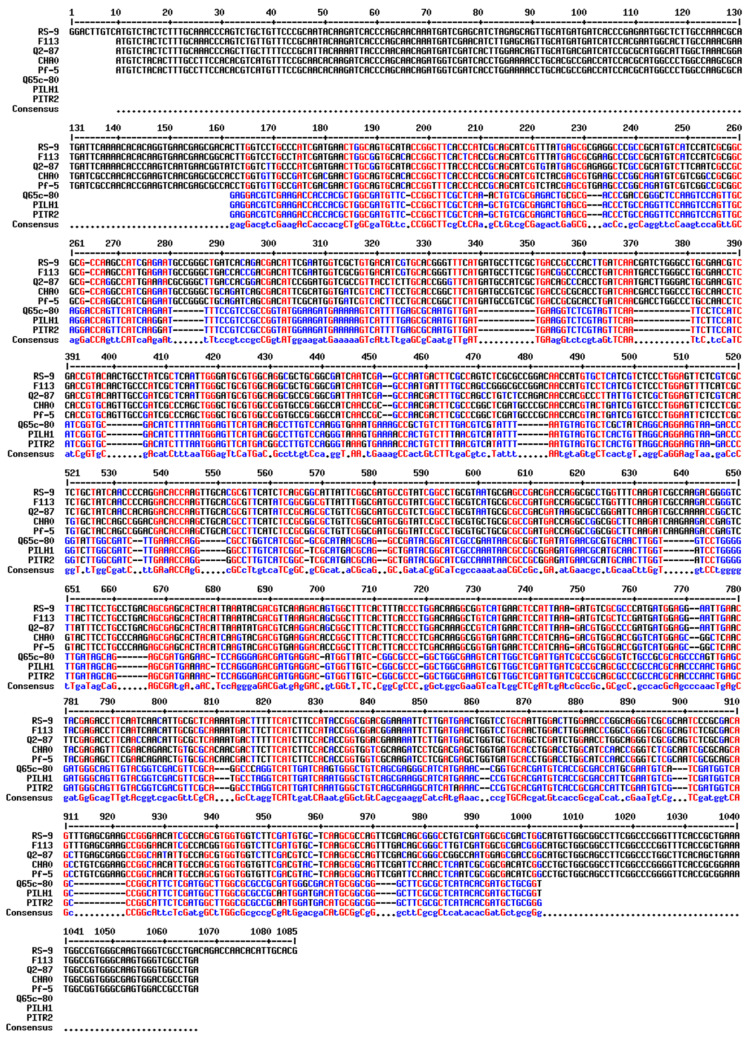
Pairwise sequence alignment of the cloned *phl*D gene (RS-9) with the reported DAPG producing *phl*D gene from *Pseudomonas* strains from the database (http://multalin.toulouse.inra.fr/multalin/, accessed on 15 March 2022). Our cloned *phl*D gene from the RS-9 strain showed homology with already reported *phl*D genes from *Pseudomonas* strain F113, Q2-87, CHAO, Pf-5, Q65c-80, PILH1, and PITR2.

**Figure 6:**
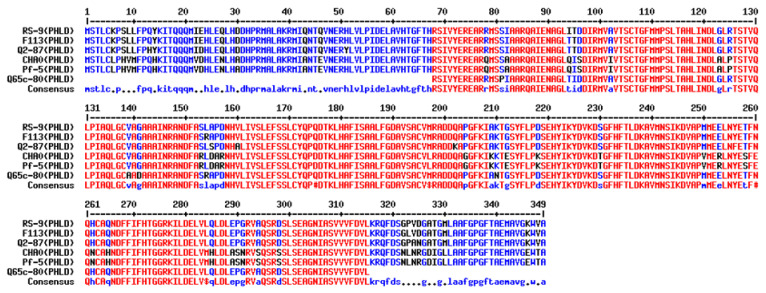
Pairwise sequence alignment of *Pseudomonas* spp. RS-9 PHLD protein with the PHLD protein from five *Pseudomonas* strains (http://multalin.toulouse.inra.fr/multalin/, accessed on 15 March 2022). The deduced amino acid sequence from the cloned *phl*D gene *Pseudomonas* strain RS-9 showed homology with the already reported PHLD protein from *Pseudomonas* strain F113, Q2-87, CHAO, and Pf-5.

Sanger sequencing of the cloned *phl*D was carried to check its identity and the results revealed that it consisted of 1078 nucleotides with an open reading frame of 1050 bp. Based on the blast results, the *phl*D gene was found to be of full-length coding for 349 amino acids. The cloned *phl*D gene showed considerable homology with the other known genes, indicating a common descent. The deduced amino acid sequence of 349 amino acids (~38.3 KDa) showed significant similarity with the homologs of PHLD (Figure 5 and Figure 6).

**Figure 7:**
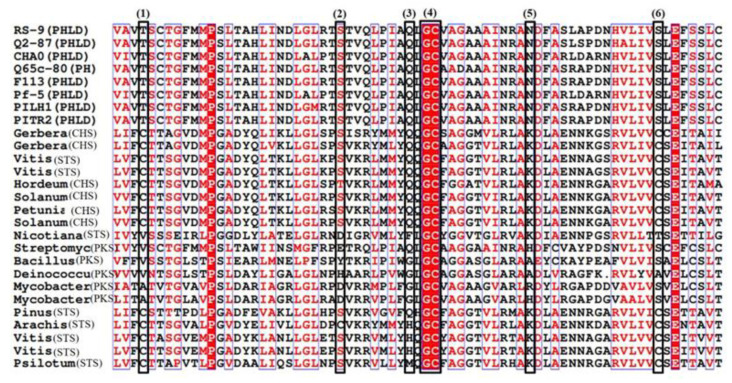
Alignment of predicted amino acid sequences for PHLD from various strains of *Pseudomonas* and type III polyketide synthase (PKS) from gram-positive bacteria and plant chalcone synthases/Stilbene synthase (CHS/STS). Conserved residues are indicated by boxes. (1) Cysteine (C135) from plant CHS implicated in substrate specificity and corresponding to threonine (T104) in PHLD. (2) Serine (S158) subunit contact site corresponding to S127 in PHLD. (3) Glutamine (Q166) residue conserved in most plant CHSs and corresponding to Q166 in the PKS of *Streptomyces griseus* and Q135 in PHLD proteins. (4) The glycine cysteine (GC) box corresponds to the conserved region with its catalytic cysteine residue. (5) Lysine (K180) residue, which corresponds to asparagine (N149) in PHLD, conserved strictly in the plant. (6) C195 involved in the product specificity in the plant CHS.

The deduced amino acid sequence from *Pseudomonas* RS-9 was compared with type III PKSs from gram-positive bacteria and CHS/STS from plants. The functional roles of key amino acid residues found in type III PKSs/CHS/STS were found in PHLD proteins and other bacterial-type III PKSs (Figure 7), such as plant C169 (cysteine-169), responsible for the catalytic activities of plant CHSs, S158 in plants (serine-158), and Q166 in plants (glutamine-166), and were conserved in PHLD proteins. C135 (cysteine-135) and C195 (cysteine-195) played roles in substrate specificity and K180 (lysine-180), which are important for the enzymatic structure and function in plant-type III PKSs, and are replaced in the bacterial counterparts. Threonine, serine, and asparagine, respectively, replaced these amino acids in the PHLD sequences (Figure 7). 

**Figure 8:**
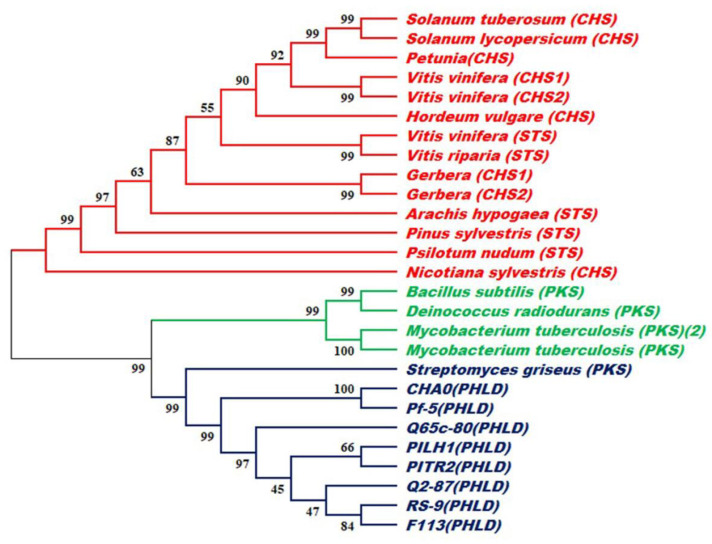
Diversity analysis between predicted amino acid sequences of PHLD from eight *Pseudomonas* strains and type III polyketide synthase from gram-positive bacteria (PKS) and plants (CHS/STS). The maximum likelihood (ML) tree was generated by the neighbor-joining method implemented in MEGA10.

Analyses of the conserved domains and annotations were performed using InterProScan. The results revealed the presence of two InterPro domains viz. the chalcone/stilbene_synthases_N-terminal domain (37-200 a.a., IPR001099) and the chalcone/stilbene_synthases_C-terminal domain (213-344 a.a., IPR012328). These two domains belong to the Polyketide_synthase_type III InterPro family (IPR011141). A maximum likelihood (ML) tree was constructed to compare the phylogenetic relation of PHLD from *Pseudomonas* to Type III PKSs from bacteria and plants (PKS from other gram-positive bacteria and CHS/STS from plants). Upon comparison, eight PHLD sequences clustered into a separate group along with PKS from *Streptomyces griseus* (PKS). PKS from gram-positive bacteria and CHS/STS from plants also clustered in a separate group in the ML tree (Figure 8).

**Figure 9:**
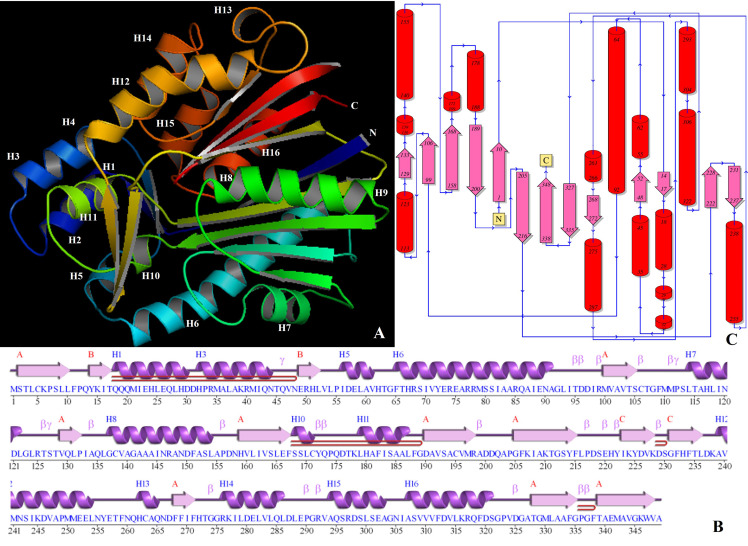
Structure of PHLD from *Pseudomonas* spp. (**A**) Stereo-ribbon diagram of the PHLD monomer (chain *A*) color-coded from the N-terminus (blue) to the C-terminus (red). Helices (H1–H16). (**B**) Diagram showing the secondary-structure elements of PHLC superimposed on its primary sequence. The labeling of the secondary-structure elements is in accordance with *PDBsum* (http://www.ebi.ac.uk/pdbsum): α-helices are labeled H1–H16, the β-strands are labeled β, β-turns and γ-turns are designated by their respective Greek letters (β, γ) and red loops indicate β-hairpins. (**C**) Topology of the PHLD protein showing the orientation of α-helices and β-strands.

The three-dimensional model of the PHLD protein using the deduced amino acid sequence of 349 amino acids was generated using the I-TASSER server (Figure 9) for the detailed structural analysis. The homology modeling of the initial structure was based on the template crystal structure of *Mycobacterium tuberculosis* polyketide synthase 11 (PKS11) (PDB entry 4JAP) [42]. The PHLD model had a C-score of 1.61 and a TM score of 0.94 ± 0.05.

**Figure 10:**
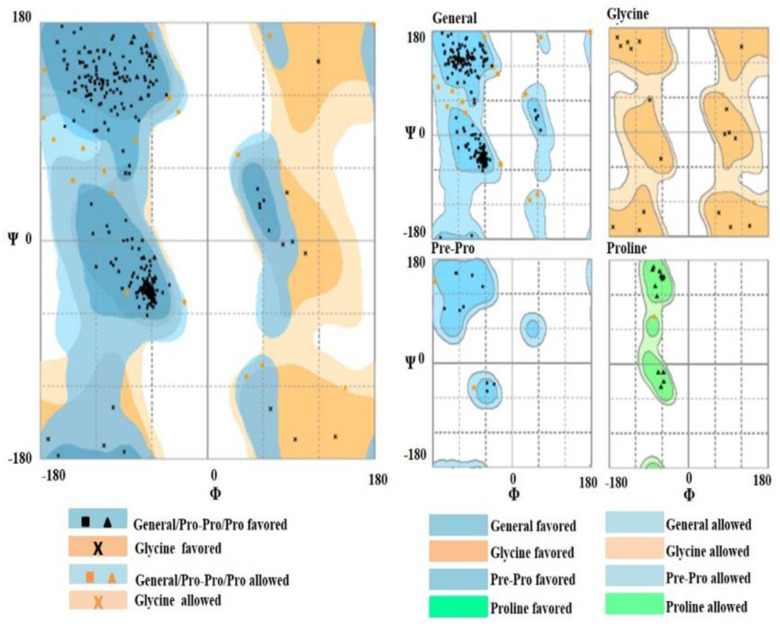
Stereochemical structure stability was analyzed using the Ramachandran plot analysis using RAMPAGE; 94.3% of residues fall in the favored region and 5.7% in the allowed region.

This initial model was refined using ModRefiner and Ramachandran plot analysis and the results of both analyses revealed that only 89.5% of residues were in the favored region, while 1% of residues were in the outlier region. The initial model was iteratively re-refined using Modloop until 0% of residues fell in the outlier region, 94.3% of residues fell in the favored region, and 5.7% in the allowed region (Figure 10). The predicted model was submitted to the Protein Model Database (PMDB) and was assigned the identifier PM0080923.

The three-dimensional model generated by homology modeling was in accordance with the secondary structure predicted by PDBsum (Figure 9B,C). The PHLD secondary structure was dominated by the presence of α-helices (40.7%) followed by β-strands (26.1%) and 3-10 helices (2.6%). The PHLD structure revealed the presence of 3 beta sheets and 16 alpha-helices. β-sheet A contained 9 mixed β-strands with topology -3X -1X 2X 1 2X 3X -1X -1, β-sheet B and C contained 2 anti-parallel β-strands with topology 1 [43]. Protein also contained 16 α-helices viz; α1 {Gln18-Gln27 (10 residues)}, α2 {Leu28-Asp30 (3 residues)}, α3{His32-Arg34 (3 residues)}, α4{Met35-Asn44 (10 residues)}, α5{Ile56-Val61 (6 residues)}, α6{Phe65-Ala91 (27 residues)}, α7{Leu114-Leu122 (9 residues)}, α8{Gly137-Val139 (3 residues)}, α9 {Ala140-Leu154 (15 residues)}, α10 {Ser168-Cys171 (4 residues)}, α11{Leu179-Leu187 (9 residues)}, α12{Ala239-Leu254 (16 residues)}, α13{His262-Gln265 (4 residues)}, α14{Arg276-Leu286 (11 residues)}, α15{Ala294-Ala303 (10 residues)} and α16{Ala307-Ser321 (15 residues)}. The 4 β-hairpins of class 29:31, 20:22, 2:2 I, and 3:3 were identified [44]. The 14 helix–helix interactions, 12 H-H types (between α1 and α4, α1, and α5, α1 and α11, α4 and α11, α6 and α7, α9, and α16, α11 and α15, α12 and α13, α12 and α14, α13 and α14, α14 and α15, and α15 and α16), and 2 H-G type (α5 and α10 and α6 and α10) were identified.

Two β-α-β motifs with 23 loops and 11 helices, and 56 loops and 42 helices participations were identified along with four β-bulges, one each of an anti-parallel classic type and anti-parallel wide type, and 2 anti-parallel G1 types were also identified. There are 21 β-turns in total belonging to 5 classes: I {(Thr95-Ile98), (Ser105-Gly108), (Tyr172-Gln175) and (Ser219-Tyr222)}; II{(Val324-Ala327) and (Gly335-Phe338)}; II’(Ile133-Leu136); IV{(Ile94-Asp97), (Ile98-Val101), (Met110-Ser113), (Arg125-Thr128), (Ala155-Asn158), (Cys171-Pro174), (Arg198-Asp201), (Leu216-Ser219), (Lys228-Gly231), (Thr273-Arg276), (Glu289-Arg292), (Pro336-Thr339) and (Gly291-Ala294)}; and VIII(His221-Lys224) [45]. Four γ turns of inverse types: (Thr45-Val47), (Met111-Ser113), (Thr126-Thr128), and (Thr273-Gly275) were also recognized.

## 4. Discussion

Special attention has been given to the antibiotic-producing fluorescent species of *Pseudomonas* due to their antibacterial [10,46], antifungal [47,48,49,50], and antiviral [51] abilities to control a wide variety of plant diseases. Advances in molecular techniques have also improved our potential to study the DAPG-producing antibacterial strains for their mechanisms of pathogen suppression and growth promotion. Breakthroughs in genomics [52,53,54,55] and transgenic [56,57] research to impart biotic/abiotic tolerance [58,59,60] or engineer traits in crops [61,62] have also driven the research in the field of biocontrol using DAPG producing strains. Genetic engineering approaches have been employed for the high-level production of phloroglucinol. Since the genetic background and metabolism of *Pseudomonas* have not been elucidated completely and the host does not respond well to genetic manipulation, the heterologous expression of *phl*D in *E. coli* is a great approach for increasing the accumulation of phloroglucinol in cultures [63,64,65].

DAPG is known to have antifungal properties and is produced by tandem activities of six genes viz. *phl*A, *phl*B, *phl*C, *phl*D, *phl*E, and *phl*F. These genes are organized as an operon onto a single nucleotide fragment of size ~ 6.5 kb. Among these six genes, *phl*D alone is important for the synthesis of MAPG. Although *phl*A, *phl*B, and *phl*C are also required for the synthesis of MAPG, *phl*D is the most essential. It has been proved that MAPG is synthesized only in the presence of *phl*D and in its absence, the cells converted exogenous MAPG to 2,4-DAPG but were unable to produce either compound themselves. This attribute makes *phl*D an important and useful marker of the genetic diversity and population structure among the 2,4-DAPG producers [14]. Thus, probes and primers specific for *phl*D have been used in combination with colony hybridization and polymerase chain reaction (PCR) to quantify the population sizes of 2,4-DAPG producers in the rhizosphere [11,66,67].

*phl*D shows a remarkable similarity to CHS/STS enzymes from plants. This is surprising because most of the microbial antibiotic enzymes are known to be synthesized via type I or type II PKSs [45,68,69,70,71]. Structural similarities between *phl*D and CHS/STS enzymes point to the common evolutionary descent and similarities in the roles they play during plant defense strongly support the instances of gene exchange between plants and bacteria [46,72]. The absence of the acyl carrier protein gene from the *phl* operon further confirms the similarity with the CHS/STS gene family. *Pseudomonas* spp. strain RS-9 was used for the isolation and cloning of the full-length *phl*D gene. The primers were designed using the *Pseudomonas fluoresens* (U41818) *phl*D gene sequence as a reference and *Pseudomonas* spp. strain RS-9 as the template. To amplify the full-length *phl* gene, we first standardized the PCR conditions. A gradient PCR was set in a temperature range of 50 °C to 60 °C to optimize the Tm for the reaction. At a lower Tm, multiple bands were obtained and at a very high Tm, faint amplification was obtained. The optimum amplification of ~1 kb for the *phl*D gene was obtained at 55 °C. This amplicon was restricted, purified, and ligated to the pBluescript vector and transformed into *E. coli* cells. 

The cloned *phl*D gene through our investigation was confirmed by Sanger sequencing and it consisted of 1078 nucleotides with an open reading frame from 10 to 1059. The longest ORF of the *phl*D gene was found to be 1050 bp. Based on the blast results, the cloned *phl*D gene was found to be full-length, coding for 349 amino acids. This is consistent with the other reports [73] (Figure 5) on the length of amino acid coding *phl* genes. The cloned *phl*D gene showed 93% homology with *phl*D genes from different *Pseudomonas* strains, such as *Pseudomonas* sp. Q12-87, *Pseudomonas* sp. K96.27, *Pseudomonas* sp. PITR2, *Pseudomonas* sp. Q37-87, *Pseudomonas* sp. 12. The deduced amino acid sequence of 349 amino acids (~38.3 KDa) showed 97% similarity with the homologs of PHLD [73]. The *phl*D gene is of utmost importance to the DAPG gene cluster as MAPG synthesis does not occur without it. 

The deduced amino acid sequence of the cloned *phl*D gene consisting of 349 amino acids was aligned pairwise with the *Pseudomonas fluorescens* (U41818) PHLD protein. The alignment revealed that there were few mutations in the protein sequence of the cloned gene. These mutations were authentic as we amplified and cloned the gene using high-fidelity Phusion *Taq* polymerase. Since this *Taq* polymerase has 3′ of proofreading activity, the chances of mis-amplifying or incorporating wrong bases are meager. These mutations need further characterization by site-directed mutagenesis. 

A comparison of the deduced amino acid sequence from *Pseudomonas* RS-9, type III PKS from plants (CHS/STS), and other gram-positive bacteria indicated that PHLD and plant CHSs displayed common features. Comparison of the active site region indicated replacements of C135, C195, and K180 with threonine, serine, and asparagine that might have influenced their substrate specificities. Lysine and asparagine codons differ only at the third nucleotide position, and a single transversion can yield an asparagine instead of a lysine. The cluster analysis clearly distinguished between PhlD and plant CHS/STS by clustering them into separate groups [74,75,76]. Type III PKS from gram-positive bacteria clustered between CHS/STS from plants and PHLD from eight *Pseudomonas* strains. PKS closest to PHLD was the *Streptomyces gresius* PKS based on cluster analysis. The possibility that type III PKSs from fluorescent pseudomonads, gram-positive bacteria, and higher plants arose independently and may represent convergent evolution of the key enzymes involved in the biosynthesis of secondary metabolites as speculated earlier [46,73], corroborating out results of the cluster analysis.

The three-dimensional structure of the PHLD protein predicted using the I-TASSER server was based on the template crystal structure of *Mycobacterium tuberculosis* polyketide synthase 11 (PKS11) (PDB entry 4JAP) [42]. The PHLD model had an excellent C-score of 1.61 indicating a good quality model. C-score ranged from −5 to 2 and this higher value indicates the high quality of the model. Similarly, a TM score >0.5 indicated a model of correct topology, and a TM score <0.17 meant a random similarity. A TM score of 0.94 ± 0.05 for PHLD indicates the precision of the predicted topology. Moreover, >90% residues (94.3% residues) in the favored region of the Ramachandran plot reaffirmed the stereochemical stability of the generated refined molecule.

## 5. Conclusions

*Pseudomonas* strains have been used as potent biocontrol agents for controlling plant diseases because of the production of metabolites with antibiotic properties [77]. Amongst them, fluorescent pseudomonads are suitable for application as biocontrol agents and are best-characterized by biocontrol PGPR [78]. The biocontrol property of *Pseudomonas* is attributed to the synthesis of phloroglucinol—a secondary metabolite with antibiotic properties—produced by genes encoded by the *phl* operon. The *phl*D gene present in the *phl* operon is singularly involved in the synthesis of MAPG, which is processed into phloroglucinol. Though *Phl*D exhibits condensing activity on malonyl CoA to produce phloroglucinol; the substrate specificity of the enzyme is not limited to malonyl CoA compared to other type III PKS enzymes. It also catalyzes “C4–C12 aliphatic acyl-CoAs and phenylacetyl-CoA” as substrates to form tri- to heptaketide pyrones [25]. The same is evidenced by the homology modelling of *Phl*D that reveals the presence of a buried tunnel that protrudes out of the active site to accommodate the binding of acyl-CoAs. Structural details revealed from our findings can be used for targeted mutagenesis and rational designs to successfully alter the substrate specificity of *Phl*D to produce derivatized products with higher potency for antibiosis. Since, *phl*D is the first committed step of DAPG biosynthesis, targeting substrate specificity of *Phl*D would be a prudent way to enhance the biocontrol activities of *Pseudomonas* spp. that otherwise are present as long-lasting indigenous communities in several agro-ecosystems to augment the capability to protect the plant root system from numerous soil-borne plant diseases. Our results of cloning and structural delineation of *phl*D will provide novel strategies for combinatorial biosynthesis of natural but pharmaceutically important metabolites with enhanced antibacterial and biocontrol effects. 

## Data Availability

Not applicable.
